# Correlation Analysis between Microscopic Pore Parameters and Macroscopic Mechanical Properties of Rock-like Materials from the Perspective of Water-Cement Ratio and Sand-Cement Ratio

**DOI:** 10.3390/ma15072632

**Published:** 2022-04-02

**Authors:** Guanglin Tian, Hongwei Deng, Yigai Xiao

**Affiliations:** 1 School of Resources and Safety Engineering, Central South University, Changsha 410083, China; tgl15352006270@163.com; 2 Sinosteel Maanshan General Institute of Mining Research Co., Ltd., Maanshan 243000, China

**Keywords:** water-cement ratio, sand-cement ratio, rock-like material, micro-pore structure, fractal dimensions, mechanical property

## Abstract

To explore the effects of water-cement ratio and sand-cement ratio on micro-pore structure characteristics and macroscopic mechanical properties and thus improve the understanding of rock-like materials, the mechanical test and detection of micro-pore structure combining NMR and SEM were carried out. The effects of WCR and SCR on different porosity parameters and mechanical properties were discussed. The correlation and internal relationship between mechanical properties and parameters of different porosities and fractal dimensions were analyzed. Experimental results showed that the different porosity parameters and fractal dimensions increased with the increase in WCR. 1.0 (SCR) was the turning point of different porosity parameters and fractal dimensions. When the SCR was less than 1.0, the porosity parameters and fractal dimension gradually decreased, while when the SCR was greater than 1.0, the porosity parameters and fractal dimension gradually increased. Microscopic porosity parameters and fractal dimension played an important role in the influence of experimental factors on mechanical properties. Different porosity parameters and fractal dimensions were negatively correlated with mechanical properties. Compressive strength and different porosity parameters conformed to a good exponential relationship, while the fitting relationship between tensile strength and mechanical properties was not obvious. This study can provide a reference for the follow-up study of rock-like materials.

## 1. Introduction

In natural geotechnical engineering, after long-term complex geological processes, there are unevenly distributed pores, microcracks, and surface fractures with different shapes inside and outside the engineering rock mass. In practical engineering activities, these microcracks and fractures have an important impact on the safety and stability of field engineering. However, due to the complexity of fracture distribution on the surface of natural engineering rock mass, the production cost of fracture samples is high, and the preparation operation is difficult, which is not conducive to the smooth progress of the test. Rock-like material is a kind of composite material with aggregates wrapped in cementitious products through complex chemical reactions of cementitious materials. It can not only simulate the mechanical properties of engineering original rock but also simulate complex fractured rock mass distributed in real engineering through prefabricated fractures. At the same time, the sample operation difficulty is low. There are broad resources, low cost, and safety in raw materials. Many studies [1,2,3,4] have shown that WCR and SCR have certain effects on the micro-pore structure and macroscopic mechanical properties of porous materials such as concrete and backfill. Therefore, to enhance the understanding of rock-like materials and the accuracy and rationality of the test, it is necessary to study the effects of WCR and SCR on the micro-pore structure and mechanical properties of rock-like materials.

Piasta [5], Li [6], Rahmanzadeh [7] discussed the effects of WCR on the compressive strength of concrete material. The studies showed that a lower WCR reduced the porosity of the transition interface between slurry and aggregate, leading to higher compressive strength. Porosity was an important factor influencing the compressive strength of WCR. Zheng [8] studied the influence of WCR on the development of compressive strength and the evolution of the micro-pore structure of cement slurry based on CT scanning and numerical modeling. The experiment results showed that the uniaxial compressive strength showed a decreasing trend with the increase in WCR, while the porosity showed a gradual increase. Kondraivendhan [9] discussed the internal relationship between the compressive strength of cement and the WCR, establishing the compressive strength calculation expression based on parameters of internal micro-pore structure. Pham [10], Consoli [11], Tariq [12] conducted experimental studies on the influence of WCR and cement admixture on the mechanical properties of cement-soil mixture. The conclusion showed that the mechanical strength was not only affected by the porosity but also depended on the value of the WCR. The compressive strength of the material gradually increased with the increase in WCR.

In the study of SCR for cementitious materials, Liu [13] carried out an experimental study on the influence of SCR on the mechanical properties of cement mortar and established a prediction model of compressive strength based on gray correlation theory. Elaqra [14] used acoustic emission, X-ray tomography, and MIP to analyze the damaging evolution of samples in different conditions of SCR in the process of loading compression. The study showed that the pore size distribution with different conditions of SCR presents different laws, and the porosity decreases with the increase in SCR. Kang [15] discussed the influence of sand size and SCR on the strength of interface bonding in cement mortar. The results found that the strength of interface bonding also gradually increased with the increase in sand size and SCR.

Based on the above studies, it can be seen that the micro-pore structure played an important role in the influence of WCR and SCR on mechanical properties. In recent years, many scholars have carried out a series of studies on the internal relationship between micro-pore structure and mechanical properties. Many researchers [16,17,18] have used different detection methods to explore the micro-pore structure. Zhang [19], Deng [20], Li [21,22,23,24] discussed the evolution of the micro-pore structure of sandstone and granite samples under freeze-thaw cycles through NMR, establishing the relationship between porosity and mechanical properties. Bu [25] carried out MIP to detect the pore volume and pore size distribution of concrete specimens with different proportions. It is proposed that statistical models of mechanical strength and micro-pore structure such as porosity and pore size distribution. Hu [26] obtained the microstructure of backfill materials by SEM and discussed the influence of additives on the pore structure and mechanical strength based on fractal theory. Qin [27] discussed the relationship between permeability and pore structure and compressive strength of silica fume concrete based on MIP and SEM. Zhang [28] carried out MIP and X-ray diffraction tests on compacted concrete to analyze the characteristics of the internal micro-pore structure and fractal dimensions. Hazra [29] took local shale in India as the main research object and systematically analyzed and discussed the internal correlation between thermal maturity and fractal dimension and pore structure.

Based on the influence of WCR and SCR on pore structure and mechanical properties, it can be seen that some studies only analyzed the influence of WCR and SCR on different scale parameters under a specific condition, but do not discuss whether the same influence law exists under different conditions. Moreover, the correlation analysis between microscopic pore parameters and macroscopic mechanical properties was still lacking. In addition, when analyzing the internal correlation between parameters of different scales, most scholars used a single detection method to test pore parameters, rather than a multi-method combined test method. The accuracy of pore structure test results is not verified, which is easy to cause experimental errors. In this paper, the microscopic detection method combining NMR and SEM was used to obtain the micro-pore structure of rock-like materials under different conditions of WCR and SCR, and the uniaxial compressive strength and tensile strength were obtained through the indoor mechanical test. The effects of WCR and SCR on porosity parameters of different radii and fractal dimensions under different conditions were analyzed, and the internal relationship between mechanical properties and pore parameters and fractal dimension was discussed, which can provide a reference value for the subsequent experimental research of rock-like materials.

## 2. Experimental Procedures

### 2.1. Experimental Materials and Schemes

Based on the current research foundation of rock-like materials, Portland cement (P.O 42.5) from a broad range of sources was selected as a cementitious material. Quartz sand with a particle size of 0.5–1 mm was selected as the aggregate, whose approximate spherical shape and smooth texture can be fully wrapped by the cementitious material. Pure silica powder and naphthalene superplasticizer were selected as admixtures, and the test solution was tap water. The specific parameters of raw materials are shown in Table 1 and Table 2. In the test, WCR and SCR were set as variable factors. The parameter of ADR was set as a constant factor. A total of 18 matching schemes were designed to analyze the influence of WCR and SCR on the micro-pore structure and mechanical properties under different proportion conditions. Among them, each group of samples contained 2 batches (3 samples in each batch), which were respectively used to obtain the compressive strength (diameter: 50 mm, height: 100 mm) and tensile strength (diameter: 50 mm, height: 50 mm). The experimental matching scheme is shown in Table 3.

### 2.2. Sample Making and Testing

First of all, following the design of matching schemes, cement, quartz sand, pure silica powder, naphthalene superplasticizer, and water solution were weighed in turn. Different raw materials were mixed and stirred. Secondly, raw materials that had been stirred were loaded into the cylindrical molds that had been prepared, placed on a vibrating table in the laboratory to vibrate until the slurry appeared on the surface of the sample. Finally, all specimens that had finished vibration were stood for 2 days and nights and then numbered after demoulding successively. They were placed into a standard curing box with a temperature of 22 °C and relative humidity of 98% for curing for 28 days.

The test was divided into two parts: micro-pore structure test and macroscopic mechanical test. The microscopic tests included NMR (Suzhou Niumag Analytical Instrument Corporation, Suzhou, China) and SEM (Changsha Institute of Mining Research Co., Ltd., Changsha, China). The macroscopic mechanical test included the uniaxial compression test and tensile test. In NMR, vacuum saturation should be performed before the test to obtain a complete pore structure inside the sample. In the mechanical test, the compressive strength and tensile strength were obtained by the microcomputer-controlled pressure testing machine: WHY-300, which was tested by Shanghai Hualong Test Instrument (ShangHai, China). According to the standard specifications of the test procedures [30], the control mode of the testing machine can be controlled by force with a loading rate of 1 KNs^−1^. Calculation formulas of compressive strength and tensile strength are as follows:

(1) $$f_{cc}=\frac{F}{A}$$

(2) $$\sigma_{t}=\frac{2P}{\pi DH}$$

**Figure 1:**
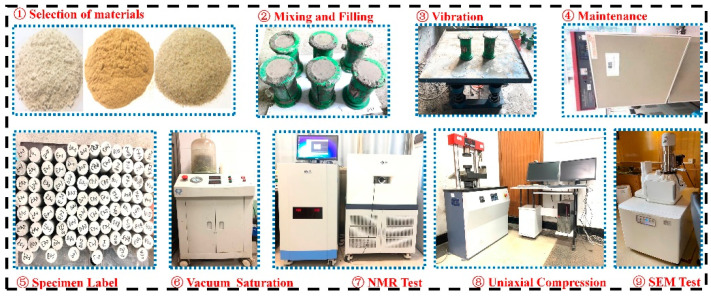
Specimen making and testing.

In formulas, $f_{cc}\;\left(\mathrm{MPa}\right)$ is the compressive strength. F(N) is the failure load of the sample and $A\left({\mathrm{mm}}^{2}\right)$ is the bearing area of the sample. $\sigma_{t}\left(\mathrm{MPa}\right)$ is the tensile strength. P(N) is the failure load. $D({\mathrm{mm}}^{2})$ is the diameter of the specimen and H(mm) is the height. Finally, the central block sample of damaged specimens was scanned by electron microscope. The whole process of the test was shown in Figure 1.

### 2.3. Pore Radius Dividing and Porosity of Different Radii Calculating

According to the principle of NMR analysis [22], the surface relaxation of internal pore water of rock-like materials can be expressed by Formula (3):

(3) $$\frac{1}{T_{2}}=\rho_{2}\frac{S}{V}$$

In the Formula (3), $T_{2}$ is the relaxation time detected by the NMR instrument. $\rho_{2}$ is the surface relaxation strength, and its value mainly depends on the rock mineral composition and internal pore surface properties. S and V are pore surface area and pore volume, respectively. Pore shape is often simplified as spherical pore, then Formula (3) can be expressed as:

(4) $$\frac{1}{T_{2}}=\rho_{2}\frac{F_{s}}{r_{c}}$$

In the Formula (4), $F_{s}$ is the pore shape factor (spherical pore, $F_{s}$ = 3) and $r_{c}$ is the pore radius. Since both $\rho_{2}$ and $F_{s}$ are constants, Formula (4) can also be expressed as:

(5) $$r_{c}=CT_{2}$$

**Figure 2:**
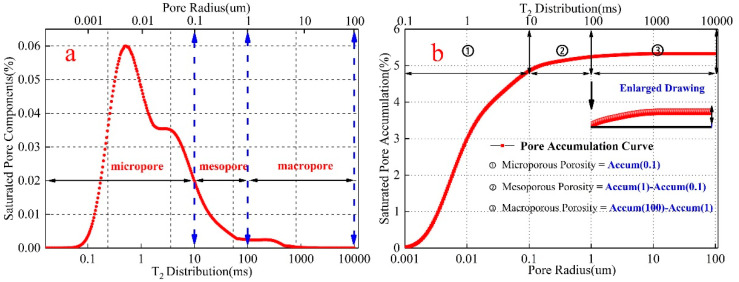
The pore size division of rock-like materials and the porosity division of different pore sizes. (**a**) shows the pore division of different radii. (**b**) shows the porosity division of different radii.

According to Formula (5), there is a linear relationship between the pore radius and relaxation time, and $T_{2}$ corresponds to $r_{c}$ one by one. That $T_{2}$ distribution detected by NMR is also the pore radius distribution. Among them, the value of C has a direct impact on the conversion of pore radius. Based on current research results on the division of pore radius in cement-based materials [31,32], the C value selected is 0.01 μm/ms. In the study of the division of micro-pore radius in rock, many scholars have put forward various methods. Referring to Zhang [19] and Yan [33]’s method of division of pore radius, this paper divided the internal pore radius into three types: micropore (pore radius < 0.1 μm), mesopore (0.1 μm ≤ pore radius ≤ 1 μm), and macropore (pore radius > 1 μm). Specific division results are shown in the Figure 2a.

Figure 2b shows the variation trend of saturated pores in rock-like materials detected by the NMR device. The red curve is the accumulated value of saturated pores. With the passage of relaxation time ($T_{2}$), the number of pores gradually increases, and the cumulative value of saturated pores also increases. Finally, the cumulative value reaches the maximum value until the pores of different radii are tested. At this time, the cumulative value is the porosity of the rock sample. According to the division of pore radius, the relaxation time corresponds to the pore radius one by one. Since the accumulation curve also changes with the change of relaxation time, accumulation values in different intervals of relaxation time are also in different intervals of pore radius. Therefore, the total porosity was divided into microporous porosity (pore radius < 0.1μm), mesoporous porosity (0.1 μm < pore radius < 1 μm) and macroporous porosity (1 μm < pore radius < 100 μm). The calculation formula is as follows:

(6) $$\{\begin{array}{c} P_{\mathrm{microp}}={\mathrm{Accum}}_{\left(0.1\right)}-{\mathrm{Accum}}_{\left(0\right)}\\ P_{\mathrm{mesop}}={\mathrm{Accum}}_{\left(1\right)}-{\mathrm{Accum}}_{\left(0.1\right)}\\ P_{\mathrm{macrop}}={\mathrm{Accum}}_{\left(100\right)}-{\mathrm{Accum}}_{\left(1\right)} \end{array}$$

In the Formula (6), $P_{\mathrm{microp}}$, $P_{\mathrm{mesop}}$ and $P_{\mathrm{macrop}}$ are microporous, mesoporous and macroporous porosity, respectively. ${\mathrm{Accum}}_{\left(0.1\right)}$, ${\mathrm{Accum}}_{\left(1\right)}$ and ${\mathrm{Accum}}_{\left(100\right)}$ are cumulative values when pore radii are 0.1, 1 and 100.

Among many methods of fractal dimension characterization of porous materials, the box dimension has the obvious advantages of a simple calculation procedure, low difficulty in use, and high precision in calculation [27,34]. Based on electron microscope photos of sample sections under different WCR and SCR conditions, the fractal dimension was calculated by using the operation program of box fractal dimension in MATLAB (2017), and the calculation formula is as follows [27]:

(7) $$D_{s}=\underset{r\to 0}{\lim}\frac{\log N_{\left(r\right)}}{\log\left(1/r\right)}$$

In the formula, $D_{s}$ is the fractal dimension. r is the side length of the square box covering the graph, and $N_{\left(r\right)}$ is the number of square boxes covering the whole graph completely. According to the calculation principle of box dimension, the smaller the side length of a square box, the more square boxes needed to cover the graph. During the calculation, logarithmic operations are performed on 1/r and $N_{\left(r\right)}$, respectively. The linear fitting is performed on the calculated logarithmic coordinates. The absolute value of the slope of the fitted line is the fractal dimension.

## 3. Result and Discussion

### 3.1. Effect of WCR on Micro-Pore Structure

**Figure 3:**
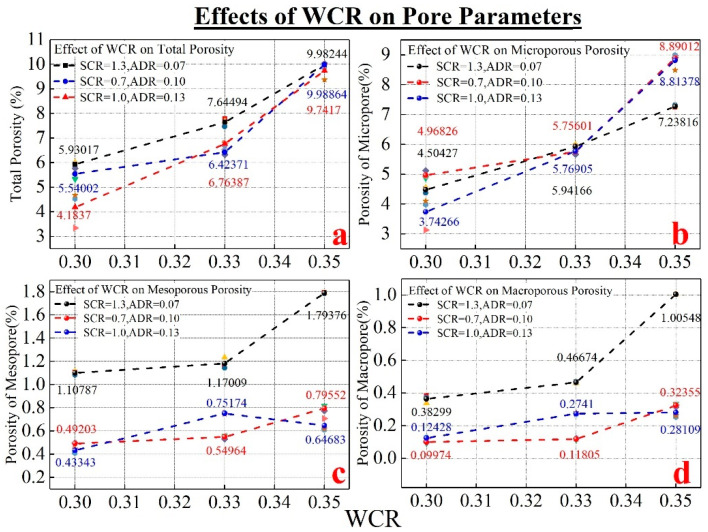
The influence of WCR on porosities of different radii. (**a**) shows the effect of WCR on the porosity. (**b**) shows the effect of WCR on the microporous porosity. (**c**) shows the effect of WCR on the mesoporous porosity. (**d**) shows the effect of WCR on the macroporous porosity.

**Figure 4:**
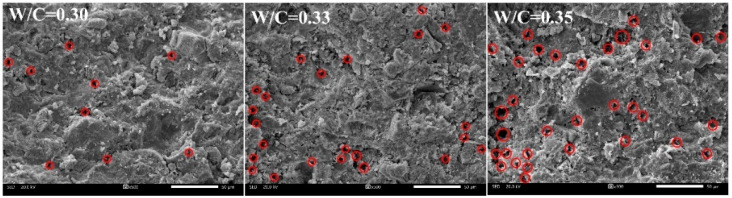
The internal microscopic characteristics magnified 500 times under different conditions of WCR.

Figure 3 and Figure 4 show porosity variation and microstructure characteristics under different conditions of WCR, respectively. It can be seen in Figure 3a, that under different SCR and ADR conditions, internal porosity shows an increasing trend with the increase in WCR. The electron microscope photo with 500 times magnification in Figure 4 also shows this variation pattern. According to the analysis in Figure 4, when WCR increased gradually, the number of pores in the material increased. The distribution morphology also became more and more abundant. It can be seen from Figure 3b–d, that the micro-pore structure was dominated by micropores and followed by mesopores. The least is macropores. Even under different SCR and ADR conditions, except for a few samples, the porosity of micropores, mesopores and macropores increased with the increase in WCR, but the degree of increase in different porosity was slightly different. Among them, the porosity of micropore increased the most, with an average increase of 3.927%. The porosity of mesopore and macropore increased slowly with an increase of 0.401% and 0.34%, respectively. In other words, the influence of SCR on the micropore was significant, while that of the mesopore and macropore was relatively low.

According to Table 2, the main components of cementable materials (P.O42.5) in production materials are different kinds of silicates. In the production process, different silicates had complex chemical reactions with aqueous solutions. The reaction equation is shown below [35,36]:

(8) $$2C_{3}S+6H\;\to {\;C}_{3}S_{2}H_{3}+3\mathrm{CH}$$

(9) $$2C_{2}S+4H\;\to {\;C}_{3}S_{2}H_{3}+\mathrm{CH}$$

According to the above formulas, the main product of the chemical reaction is calcium silicate hydrate ($C_{3}S_{2}H_{3}$) [37,38,39]. In the complex production process, C-S-H generated by hydration reaction can account for 60–70% of the total number of hydration products [40]. However, C-S-H is a porous material with a pore radius of 0.5~1 nm, which is composed of multiple C-S-H slices and 18% nanoporosity [41]. With the increase in WCR, the degree of hydration reaction between silicate and water also increased, which promotes the formation of C-S-H, finally leading to the increase in microscopic porosity.

In addition, free water in hydration products was another factor leading to the increase in porosity of rock-like materials with the increase in WCR. During hydration, C-S-H occupied the original water-filled space and gradually aggregated around the unhydrated cement, eventually forming a hardened material consisting of hydration products wrapped around the aggregate and anhydrous cement particles. However, when the water in the subsequent water-filled area evaporated, there were no hydration products to fill it. So, pores of various shapes began to form inside the material in the late hydration stage. In summary, as the WCR increased continuously, more free water would remain in the material after removing the mass of the water solution required to participate in the hydration reaction. Then, this free water evaporated later to form more microscopic pores, so the experimental phenomenon was that porosity increased with the increase in WCR.

### 3.2. The Influence of Water-Cement Ratio on Fractal Dimension

**Figure 5:**
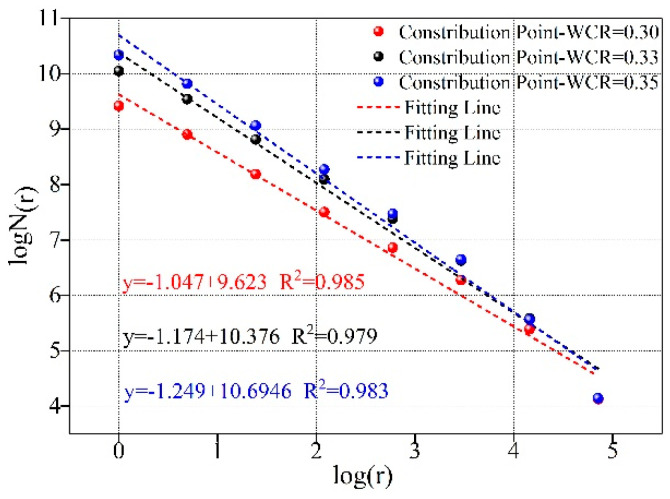
Fractal dimension calculation of rock-like materials under different WCR.

**Figure 6:**
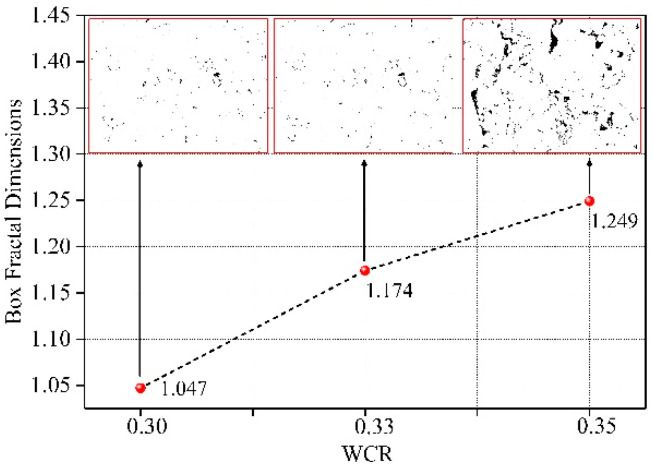
The influence of water-cement ratio on fractal dimension.

Figure 5 shows the calculation results of fractal dimensions under different conditions of WCR. It can be seen from the figure that the fractal dimensions under different experimental conditions were all greater than 1. The fitting relationship of fractal dimensions under each test condition was greater than 0.975. The spatial distribution of microscopic pores in rock-like materials under different conditions of WCR had fractal characteristics in a certain range. Figure 6 shows the influence rule of WCR on fractal dimension. As can be seen from the figure, with the continuous increase in WCR, the number of microscopic pores increased. The pore morphology distribution became more and more irregular. The fractal dimension also became larger and larger. Combined with the above influence law of WCR on porosity, it can be seen that the increase in WCR led to the increase in the number of microscopic pores. The increase in porosity led to the increase in the fractal dimension. The more pores that were developed, the larger the fractal dimension was. The experimental data showed that the pore distribution under different conditions had a good self-similarity. The box fractal dimension can be used to characterize the pore distribution characteristics.

### 3.3. The Influence of SCR on Micro-Pore Structure

**Figure 7:**
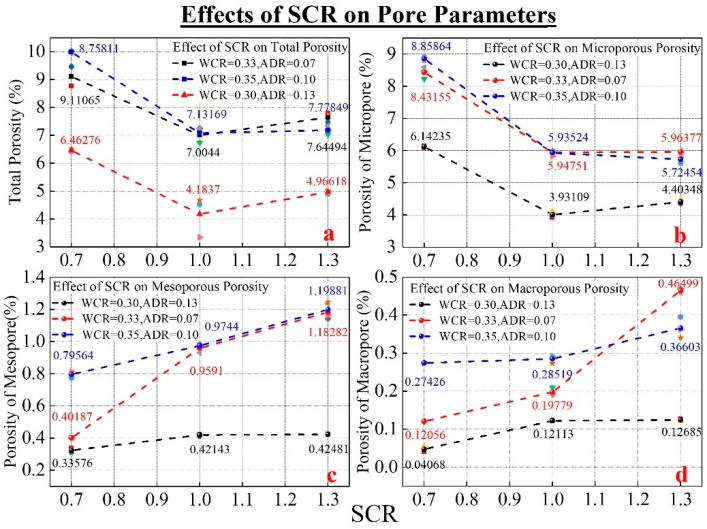
The influence of SCR on microporous, mesoporous, and microporous porosity. (**a**) shows the effect of SCR on the porosity. (**b**) shows the effect of SCR on the microporous porosity. (**c**) shows the effect of SCR on the mesoporous porosity. (**d**) shows the effect of SCR on the macroporous porosity.

**Figure 8:**
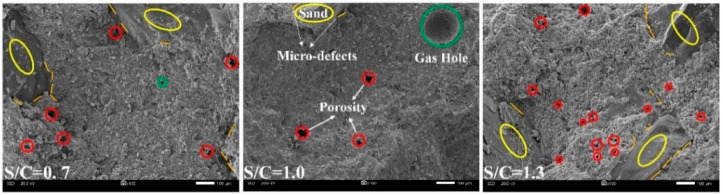
Microstructure of rock-like materials with different SCR.

Figure 7 and Figure 8 show the change rule and internal microscopic characteristics of porosity parameters under different conditions of SCR, respectively. It can be seen in Figure 7 that the increase in quartz sand had a significant impact on the porosity of different radii. Although under different conditions of WCR and ADR, the impact of SCR on different porosity had the same change. When SCR increased from 0.7 to 1.3, different porosity parameters decreased first and then increased. Figure 8 shows the microscopic characteristics of electron microscope magnification of 100 times. According to Figure 8, it can be seen that the main composition of the sample included hydration products, quartz sand, pores of different radii, and micro-defects. The pores mainly exist in hydration products. The micro-defects were distributed at the interface between hydration products and quartz sand. Compared with the electron microscope figure of 0.7 and 1.3, it can be found that the amount of quartz sand was significantly increased. The increase in the quartz sand filled part of hydration products but also produced micro-defects between hydration products and quartz sand.

In the variation of porosity with SCR, 1.0 (SCR) was a special point. When the SCR was less than 1.0, porosity decreased with the increase in SCR. However, when SCR was greater than 1.0, porosity increased with the increase in SCR. Based on the variation law of porosity of micropores, mesopores, and macropores and the analysis of electron microscope photo, when SCR increased from 0.7 to 1.0, the increased quartz sand filled part of hydration products to improve the compactness. So, the decreasing law of porosity and micropore porosity appeared. However, the increase in the number of quartz sand also led to the formation of micro-defects. The porosity of mesopore and macropore gradually increased. When the increase in mesopore and macropore porosity cannot offset the decrease in micropore porosity, there is a decrease in porosity. As the SCR continued to increase from 1.0 to 1.3, the number of micro-defects continued to increase as the amount of quartz sand increased, leading to an increase in the porosity of mesopore and macropore. The increase in mesopore and macropore porosity offset the decrease in micropore porosity in the early stage, forming a transition from decrease to increase in porosity.

### 3.4. The Influence of SCR on Fractal Dimension

**Figure 9:**
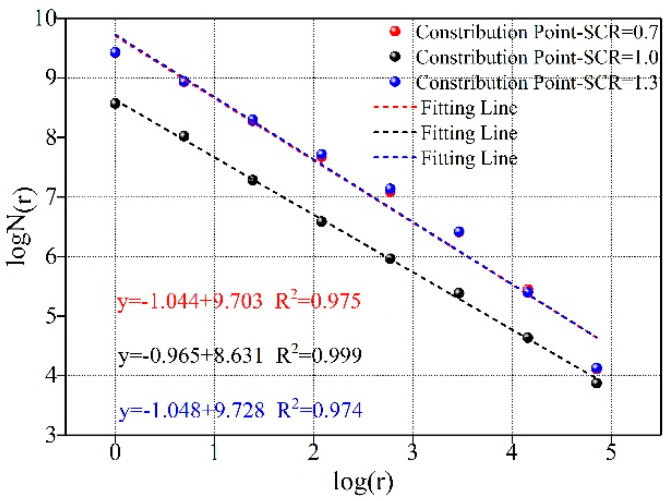
Calculation of fractal dimension under different SCR.

**Figure 10:**
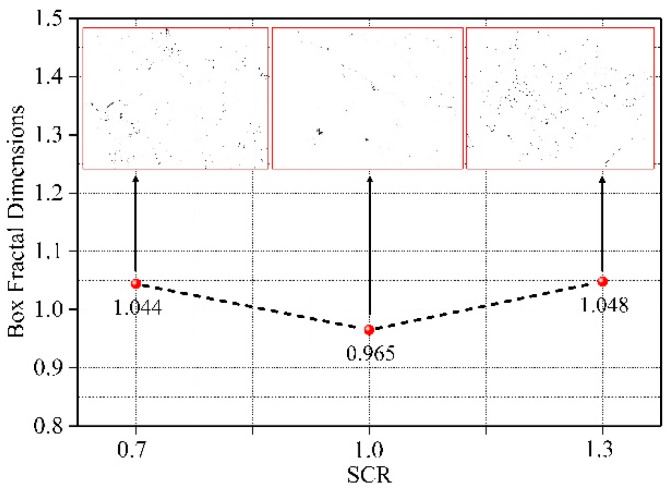
The influence of SCR on fractal dimension.

Figure 9 shows the calculation results of fractal dimensions under different conditions of SCR. According to the calculation results, the fractal dimension with SCR of 0.7 and 1.3 was greater than 1. The fractal dimension with an SCR of 1.0 was less than 1. However, the fitting relationship of fractal dimension under each condition was greater than 0.97, indicating that the spatial distribution of microscopic pores under different conditions also had fractal characteristics within a certain range. Figure 10 shows the influence rule of SCR on the fractal dimensions. It can be seen from the figure that 1.0 was also a special point in the variation trend of the fractal dimensions. When the SCR was less than 1.0, the fractal dimension showed a decreasing trend. However, when the SCR was greater than 1.0, the fractal dimension showed an increasing trend. According to the fractal theory, the more developed the pores in porous materials are. The more irregular their distribution is. The larger the fractal dimension is. Combined with the SCR on the influence of porosity, when SCR increased from 0.7 to 1.0, internal porosity decreased. The fractal dimension also showed a trend of decline. When SCR increased from 1.0 to 1.3, the increase in micro-defects led to the continuous increase in the number of pores and the increasingly irregular distribution. The fractal dimension also presented an increasing trend.

### 3.5. Correlation Analysis between Mechanical Properties and Porosity and Fractal Dimension

**Figure 11:**
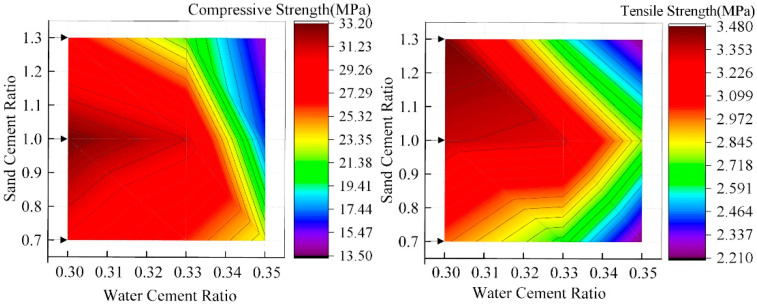
The influence of WCR on compressive strength and tensile strength.

**Figure 12:**
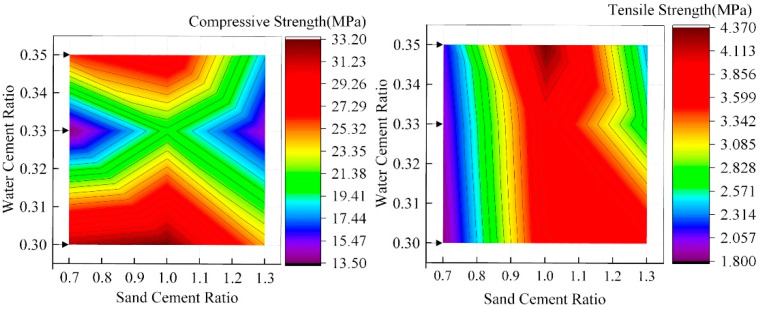
The influence of SCR on compressive strength and tensile strength.

**Figure 13:**
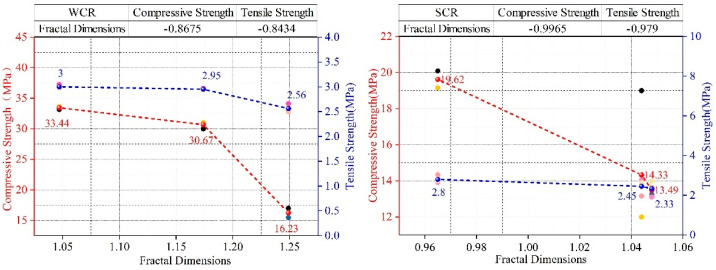
The influence of fractal dimension on compressive strength and tensile strength.

**Figure 14:**
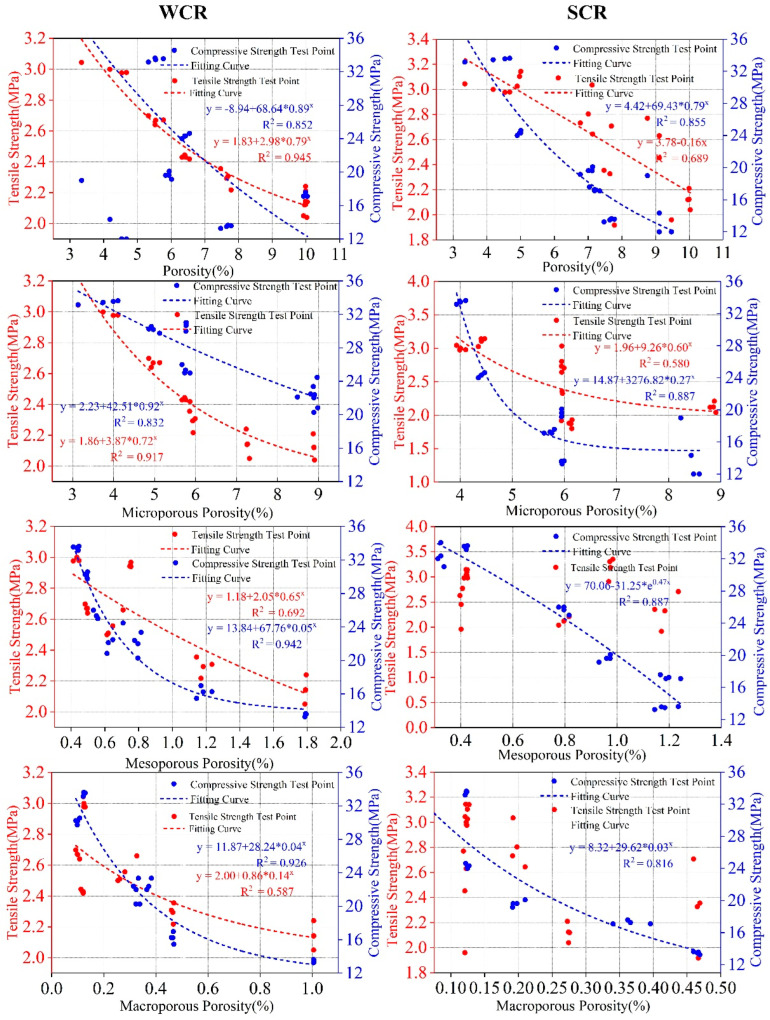
Fitting analysis of different porosity parameters and mechanical properties.

Figure 11 and Figure 12 show the influence rules of WCR and SCR on the compressive strength and tensile strength of rock-like materials, respectively. In accordance with the figure, although under different conditions of SCR, the compressive strength and tensile strength gradually decreased with the continuous increase in WCR. However, in the influence law of SCR on mechanical properties, 1.0 (SCR) was the special point. Although the specimens were in different conditions of WCR, when SCR was less than 1.0, the compressive strength and tensile strength increased gradually with the increase in SCR. When SCR was greater than 1.0, the mechanical properties decreased gradually with the increase in SCR. Combined with the above influence laws of WCR and SCR on different porosity parameters and fractal dimensions, it can be seen that WCR and SCR have corresponding influence laws on porosity parameters with different radii and fractal dimensions. In order to verify whether there is an internal correlation between microscopic porosity parameters and macroscopic mechanical properties, correlation coefficient calculation and fitting analysis between mechanical properties and porosity parameters and the fractal dimension were carried out. The calculation results are shown in Table 4, Figure 13 and Figure 14.

The correlation coefficient is the correlation between two variables in quantitative correlation analysis, usually represented by “r”, whose calculated value range is [−1, 1]. When r is a positive value, the correlation is positive. The values of the two variables increase together. When r is a negative value, the correlation is negative. When the value of one variable increases, the other variable will gradually decrease. In the analysis of the correlation of two variables, the correlation coefficient is divided into four grades: the weak correlation (0.2 ≤ |r| ≤ 0.4), the moderate correlation (0.4 ≤ |r| ≤ 0.6), the strong correlation (0.6 ≤ |r| ≤ 0.8), the extremely strong correlation (0.8 ≤ |r|≤ 1.0). Figure 13 shows the influence of fractal dimension on mechanical properties. It can be seen that the correlation between the fractal dimension, compressive strength, and tensile strength showed a good negative correlation regardless of the WCR and SCR, which was above 0.84. By increasing the fractal dimension, the compressive strength and tensile strength decreased.

According to Table 4, under both WCR and SCR, the correlation between different porosity parameters and mechanical properties is negative. However, the correlation coefficient between porosity parameters and compressive strength is higher than that between porosity parameters and tensile strength. The correlation coefficient between pore porosity and compressive strength is higher than 0.85, except that the correlation coefficient between pore porosity and compressive strength is lower than 0.80 under the condition of SCR. However, in the correlation coefficient between tensile strength and different porosity parameters, the correlation coefficient of some test conditions is even lower than 0.5. According to the analysis of the fitting results of porosity parameters and mechanical properties (Figure 14), whether the conditions are WCR or SCR, different porosity parameters are in a good exponential relationship with the compressive strength. The fitting correlation coefficients are more than 0.80. The compressive strength shows an exponentially decreasing trend with the increase in porosity parameters. However, in the fitting analysis of tensile strength and porosity parameters, the fitting effect is not obvious. Most of the correlation coefficients are below 0.70. Among them, the porosity of mesopore (−0.2604) and macropore (−0.5716) with a small correlation coefficient do not even fit the tensile strength well.

Based on the correlation coefficients and fitting results above, porosity parameters played an extremely important role in the influence of WCR and SCR on mechanical properties. The variation of experimental conditions affected the distribution and quantity of microscopic pores. When the number of internal pores in the material gradually increased, the pores expanded during the loading process of the sample, and different pores would be connected, accelerating the rupture and ultimately leading to the decline of mechanical properties.

## 4. Conclusions

In this paper, the porosity parameters of different radii and fractal dimensions of rock-like materials under WCR and SCR were obtained by using the microscopic pore structure detection method combined with NMR and SEM. The effects of WCR and SCR on porosity, fractal dimension, and mechanical properties were analyzed, and the internal relationship between mechanical properties and porosity parameters, and fractal dimension was discussed. The main conclusions are as follows:

1. Although under different experimental conditions of SCR, WCR had the same effect on porosity parameters and fractal dimensions with different radii. With the increase in WCR, porosity parameters and fractal dimension of micropores, mesopores, and macropores showed an increasing trend.
2. 1.0 was the special point where SCR influenced the porosity with different radii and fractal dimensions. Although under different experimental conditions of WCR, when the SCR was less than 1.0, the porosity parameters of different radii and fractal dimensions decreased with the increase in SCR. When the SCR was greater than 1.0, the porosity parameters of different radii and fractal dimensions increased gradually with the increase in SCR.
3. Different porosity parameters played important roles in the influence of WCR and SCR on the mechanical properties. The porosity parameters of different radii were negatively correlated with tensile strength and compressive strength, but the correlation coefficient between porosity parameters and compressive strength was significantly higher than that between tensile strength and porosity parameters. Different porosity parameters had good exponential relationships with the compressive strength, but the fitting effect with the tensile strength was not obvious. The compressive strength decreased exponentially with the increase in porosity parameters.
4. The fractal dimension was negatively correlated with tensile strength and compressive strength. Both tensile strength and compressive strength decreased with increasing fractal dimensions.

## Figures and Tables

**Table 1:** Chemical composition of Portland cement.

Chemical Composition	3CaO·SiO_2_	2CaO·SiO_2_	3CaO·Al_2_O_3_	4CaO·Al_2_O_3_·Fe_2_O_3_
Content	52.8%	20.7%	11.5%	8.8%

**Table 2:** Detailed parameters of rock-like material.

Material	Traits	Main Ingredients	Particle Size	Density (g/cm^3^)
Quartz sand	Yellow and white particles	Quartz > 95%	0.5–1.0 mm	1.49
Silica fume	White powder	SiO_2_ > 99%	1 μm	2.2–2.6
Naphthalene water reducer	Brown yellow powder	β-Naphthal-enesulfonate sodium formaldehyde condensate	-	-

**Table 3:** Experimental schemes.

Exerimental Scheme	WCR	SCR	ADR	Exerimental Scheme	SCR	WCR	ADR
1	0.30	0.7	0.10	4	0.7	0.30	0.13
	0.33				1.0		
	0.35				1.3		
2	0.30	1.0	0.13	5	0.7	0.33	0.07
	0.33				1.0		
	0.35				1.3		
3	0.30	1.3	0.07	6	0.7	0.35	0.10
	0.33				1.0		
	0.35				1.3		

**Table 4:** Correlation coefficients between different porosity and mechanical properties.

Parameter Type	WCR		SCR	
	Compressive Strength (MPa)	Tensile Strength (MPa)	Compressive Strength (MPa)	Tensile Strength (MPa)
Porosity	−0.9438	−0.9146	−0.9058	−0.8306
Microporous Porosity	−0.9097	−0.9035	−0.7784	−0.6893
Mesoporous Porosity	−0.8869	−0.8289	−0.9704	−0.2604
Macroporous Porosity	−0.8666	−0.7436	−0.8853	−0.5716

## Data Availability

The data presented in this study are available on request from the corresponding author.

## Abbreviations

Symbols & Notations	
WCR	Water-cement ratio
SCR	Sand-cement ratio
ADR	Admixture
NMR	Nuclear Magnetic Resonance
SEM	Scanning Electron Microscope
MIP	Mercury Injection Experiment
CT	Computed Tomography
$C_{3}S$	3CaO·SiO_2_
$C_{2}S$	2CaO·SiO_2_
H	H_2_O
$C_{3}S_{2}H_{3}$	3CaO·SiO_2_·3H_2_O

## References

[1] Jiang C., Guo W., Chen H., Zhu Y., Jin C. (2018). Effect of filler type and content on mechanical properties and microstructure of sand concrete made with superfine waste sand. Constr. Build. Mater..

[2] Diambra A., Festugato L., Ibraim E., da Silva A.P., Consoli N.L. (2018). Modelling tensile/compressive strength ratio of artificially cemented clean sand. Soils Found..

[3] Jueyendah S., Lezgy-Nazargah M., Eskandari-Naddaf H., Emamian S. (2021). Predicting the mechanical properties of cement mortar using the support vector machine approach. Constr. Build. Mater..

[4] Chuta E., Colin J., Jeong J. (2020). The impact of the water-to-cement ratio on the surface morphology of cementitious materials. J. Build. Eng..

[5] Piasta W., Zarzycki B. (2017). The effect of cement paste volume and w/c ratio on shrinkage strain, water absorption and compressive strength of high performance concrete. Constr. Build. Mater..

[6] Li L., Zhang H., Guo X., Zhou X., Lu L., Chen M., Cheng X. (2019). Pore structure evolution and strength development of hardened cement paste with super low water-to-cement ratios. Constr. Build. Mater..

[7] Rahmani K., Rahmanzadeh B., Piroti S. (2018). Experimental study of the effect of water-cement ratio on compressive strength, abrasion resistance, porosity and permeability of Nano silica concrete. Frat. Integrità Strutt..

[8] Zheng S., Liu T., Jiang G., Fang C., Feng Y. (2021). Effects of Water-to-Cement Ratio on Pore Structure Evolution and Strength Development of Cement Slurry Based on HYMOSTRUC3D and Micro-CT. Appl. Sci..

[9] Kondraivendhan B., Bhattacharjee B. (2016). Strength and w/c ratio relationship of cement based materials through pore features. Mater. Charact..

[10] Pham T.A., Koseki J., Dias D. (2021). Optimum material ratio for improving the performance of cement-mixed soils. Transp. Geotech..

[11] Consoli N.C., Festugato L., da Rocha C.G., Cruz R.C. (2013). Key parameters for strength control of rammed sand–cement mixtures: Influence of types of portland cement. Constr. Build. Mater..

[12] Tariq K., Maki T. (2014). Mechanical behaviour of cement-treated sand. Constr. Build. Mater..

[13] Liu K., Li Y., Wang F., Ren J., Xie H. (2018). Modeling and experimental study of multiple factors on mechanical strength of iron sand modified cement mortars. Constr. Build. Mater..

[14] Elaqra H., Godin N., Peix G., R’Mili M., Fantozzi G. (2007). Damage evolution analysis in mortar, during compressive loading using acoustic emission and X-ray tomography: Effects of the sand/cement ratio. Cem. Concr. Res..

[15] Kang S., Kim J., Dong J., Chung Y. (2013). Effect of sand grain size and sand-to-cement ratio on the interfacial bond strength of steel fibers embedded in mortars. Constr. Build. Mater..

[16] Deng H., Duan T., Tian G., Liu Y., Zhang W. (2021). Research on Strength Prediction Model and Microscopic Analysis of Mechanical Characteristics of Cemented Tailings Backfill under Fractal Theory. Minerals.

[17] Deng H., Tian G., Yu S., Jiang Z., Zhang Y. (2020). Research on Strength Prediction Model of Sand-Like Material Based on Nuclear Magnetic Resonance and Fractal Theory. Appl. Sci..

[18] Yu S., Deng H., Tian G., Ke Y. (2022). Research on dynamic compressive characteristics of rock-like material considering the influence of crack angles and freeze–thaw cycle. Arab. J. Geosci..

[19] Zhang J., Deng H., Deng J., Gao R. (2019). Fractal Analysis of Pore Structure Development of Sandstone: A Nuclear Magnetic Resonance Investigation. IEEE Access.

[20] Deng H., Yu S., Deng J. (2018). Damage Characteristics of Sandstone Subjected to Coupled Effect of Freezing-Thawing Cycles and Acid Environment. Adv. Civ. Eng..

[21] Li J., Liu H., Kaiming A., Zhu L. (2018). An NMR-Based Experimental Study on the Pore Structure of the Hydration Process of Mine Filling Slurry. Adv. Civ. Eng..

[22] Li J., Zhou K., Liu W., Zhang Y. (2018). Analysis of the effect of freeze–thaw cycles on the degradation of mechanical parameters and slope stability. Bull. Eng. Geol. Environ..

[23] Li J., Kaunda R., Zhou K. (2018). Experimental investigations on the effects of ambient freeze-thaw cycling on dynamic properties and rock pore structure deterioration of sandstone. Cold Reg. Sci. Technol..

[24] Li J., Zhou K., Liu W., Deng H. (2016). NMR research on deterioration characteristics of microscopic structure of sandstones in freeze–thaw cycles. Trans. Nonferrous Met. Soc. China.

[25] Bu J., Tian Z. (2016). Relationship between pore structure and compressive strength of concrete: Experiments and statistical modeling. Sadhana.

[26] Hu J., Ren Q., Yang D., Ma S., Luo Z. (2020). Cross-scale characteristics of backfill material using NMR and fractal theory. Trans. Nonferrous Met. Soc. China.

[27] Lü Q., Qiu Q., Zheng J., Wang J., Zeng Q. (2019). Fractal dimension of concrete incorporating silica fume and its correlations to pore structure, strength and permeability. Constr. Build. Mater..

[28] Zhang L., Zhou J. (2020). Fractal characteristics of pore structure of hardened cement paste prepared by pressurized compact molding. Constr. Build. Mater..

[29] Hazra B., Wood D.A., Kumar S., Saha S., Dutta S., Kumari P., Singh A.K. (2018). Fractal disposition, porosity characterization and relationships to thermal maturity for the Lower Permian Raniganj basin shales, India. J. Nat. Gas Sci. Eng..

[30] (2020). SL/T 264-2020 Rock Test Regulations for Water Conservancy and Hydropower Engineering.

[31] Valckenborg R.M.E., Pel L., Hazrati K., Kopinga K., Marchand J. (2001). Pore water distribution in mortar during drying as determined by NMR. Mater. Struct..

[32] Muller A.C., Scrivener K.L., Gajewicz A.M., McDonald P.J. (2013). Densification of C–S–H Measured by^1^H NMR Relaxometry. J. Phys. Chem. C.

[33] Jian-Ping Y., Dan-Ni W., Zun-Zhi L., Bin G., Jin-Gong C., Qiang L., Yu Y. (2016). The quantitative evaluation method of low permeable sandstone pore structure based on nuclear magnetic resonance (NMR) logging. Chin. J. Geophys..

[34] Li S., Dong Q., Ni F., Jiang J., Han Y. (2018). Evaluation of Susceptibility of High-Temperature Performance of Asphalt Mixture to Morphological Feature of Aggregates by Fractal Theory. J. Mater. Civ. Eng..

[35] Papadakis V.G. (2000). Effect of supplementary cementing materials onconcrete resistance against carbonation and chloride ingress. Cem. Concr. Res..

[36] Wang X., Lee H. (2010). Modeling the hydration of concrete incorporating fly ash or slag. Cem. Concr. Res..

[37] Kondraivendhan B., Bhattachaijee B. (2010). Effect of Age and Water-cement Ratio on Size and Dispersion of Pores in Ordinary Portland Cement Paste. ACI Mater. J..

[38] Chen X., Wu S., Zhou J. (2013). Influence of porosity on compressive and tensile strength of cement mortar. Constr. Build. Mater..

[39] Zhao H., Xiao Q., Huang D., Zhang S. (2014). Influence of Pore Structure on Compressive Strength of Cement Mortar. Sci. World J..

[40] Sekkal W., Zaoui A., Benzerzour M., Abriak N. (2016). Role of porosity on the stiffness and stability of (001) surface of the nanogranular C–S–H gel. Cem. Concr. Res..

[41] Constantinides G., Ulm F. (2007). The nanogranular nature of C-S-H. J. Mech. Phys. Solids.

